# Performance and Consistency of Indicator Groups in Two Biodiversity Hotspots

**DOI:** 10.1371/journal.pone.0019746

**Published:** 2011-05-26

**Authors:** Joaquim Trindade-Filho, Rafael Dias Loyola

**Affiliations:** 1 Programa de Pós-Graduação em Ecologia e Evolução, Instituto de Ciências Biológicas, Universidade Federal de Goiás, Goiânia, Goiás, Brazil; 2 Departamento de Ecologia, Instituto de Ciências Biológicas, Universidade Federal de Goiás, Goiânia, Goiás, Brazil; University of Western Ontario, Canada

## Abstract

**Background:**

In a world limited by data availability and limited funds for conservation, scientists and practitioners must use indicator groups to define spatial conservation priorities. Several studies have evaluated the effectiveness of indicator groups, but still little is known about the consistency in performance of these groups in different regions, which would allow their *a priori* selection.

**Methodology/Principal Findings:**

We systematically examined the effectiveness and the consistency of nine indicator groups in representing mammal species in two top-ranked Biodiversity Hotspots (BH): the Brazilian Cerrado and the Atlantic Forest. To test for group effectiveness we first found the best sets of sites able to maximize the representation of each indicator group in the BH and then calculated the average representation of different target species by the indicator groups in the BH. We considered consistent indicator groups whose representation of target species was not statistically different between BH. We called effective those groups that outperformed the target-species representation achieved by random sets of species. Effective indicator groups required the selection of less than 2% of the BH area for representing target species. Restricted-range species were the most effective indicators for the representation of all mammal diversity as well as target species. It was also the only group with high consistency.

**Conclusions/Significance:**

We show that several indicator groups could be applied as shortcuts for representing mammal species in the Cerrado and the Atlantic Forest to develop conservation plans, however, only restricted-range species consistently held as the most effective indicator group for such a task. This group is of particular importance in conservation planning as it captures high diversity of endemic and endangered species.

## Introduction

The current extinction rate surpasses more than a thousand times the basal rate of fossil records [1], and it should keep rising as human use of Earth's natural ecosystems increases [2]–[4]. To curb the effects of human pressure on biodiversity, conservation scientists, practitioners and policy makers collaborate to propose and establish natural protected areas, which still stand as the most effective and least expensive conservation strategy worldwide to ensure long-term conservation of species' populations [5], [6]. However, resources available for conservation are limited, requiring planned strategies. This recognition led to the development of systematic conservation planning, which aims to ensure efficient use of scarce resources for conservation [7], [8].

Despite the impressive efforts of current research, our knowledge of biodiversity is negligible in comparison with the urgency imposed by the current biodiversity crisis [9], [10]. Constrained by data availability, conservation planners have used biodiversity surrogates when selecting sites of interest for conservation [11]–[13]. However, site-selection methods for biodiversity conservation rely fundamentally on information about the spatial distribution of biodiversity [7], which is still very limited (a problem known as the ‘Wallacean shortfall’). Moreover, available data on species' distribution are usually strongly biased to temperate and subtropical regions, as well as to particular taxonomic groups (e.g. mammals and birds). This entails a problem because lesser-known regions of the world are usually those with the greatest biodiversity, being also the regions with the greatest need for well designed and established conservation plans [1].

Conservation planning is necessarily based on biodiversity surrogates for whom data can be obtained [5], [14]. Biodiversity surrogates are usually separated into two categories: (1) surrogates based on species, being either multi species (e.g. indicator groups) or single species (e.g. keystone species, umbrella species, and ‘flagship species’) [12], [15], [16], and (2) surrogates based on biotic and abiotic features, which can be mapped (e.g. remotely-sensed vegetation, land cover and environmental gradients) [12], [17]–[20].

Surrogates based on indicator groups are substantially more effective than those based on environmental data [5]. Indicator groups can be defined as sets of species whose geographical distribution coincides with the aggregate distribution of other taxonomic groups so that their representation will ensure the representation of diversity as a whole [21], [22]. Of course, to act as an indicator group candidate groups must have known geographic distribution [11], [23], and several methods have been proposed for the selection and evaluation of indicator group effectiveness [5], [11], [21], [24]. Thus far, such evaluation has produced diverse and often contradictory results [5], [25]–[28]. These contradictions relate to the nature of biodiversity features being represented, the choice of surrogates, differences among study regions, and the method applied to quantify surrogate effectiveness [12], [29]. Therefore, it is currently impossible to make any generalization about the consistency of indicator groups, i.e. their effective performance in different geographic regions. Systematic investigations on the consistency of indicator groups would allow the selection of these groups *a priori* helping to accelerate conservation assessments as well as the decision-making process. Despite the obvious need for investigating the consistency of indicator groups, only very few studies have explicitly evaluated this aspect [11].

Here we used a biodiversity-rich data set of terrestrial mammals to systematically assess the effectiveness and consistency of indicator groups in two top-ranked Biodiversity Hotspots: the Brazilian Cerrado and the Atlantic Forest. First, we investigated the ability of each indicator group to represent all mammals, as well as endemic, threatened, and rare mammal species. Then, we assessed the consistency of indicator groups by comparing the ability of nine different sets of species to act as surrogates for all mammal species in both Biodiversity Hotspots. We show that even though more than one indicator group could be used as a surrogate for the representation of mammal biodiversity, only one of them (the restricted-range species) is consistent in its ability to represent mammals, including endemic and threatened species, in both Biodiversity Hotspots.

## Results

### Indicator group performance in representing all species

**Figure 1:**
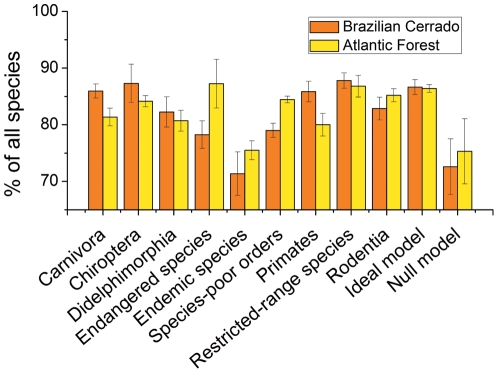
Effectiveness and the consistency of indicator groups to represent all mammal species in the Cerrado and the Atlantic Forest Biodiversity Hotspots. Effectiveness and consistency were measured as the percentage of all species represented in eight (Cerrado) and nine (Atlantic Forest) sites selected to protected all mammal species. Bars heights represent means of 20 reserve-selection analyses, error bars represent standard deviations. The ideal model and the null model stand for the result of sites selected based on all species pooled together and random species sets, respectively.

Sites selected based on different indicator groups captured more mammal species than those selected at random, in both Biodiversity Hotspots (F_21, 418_ = 73.86, p<0.01, Fig. 1 and Table S1). Endemic species did not achieve high representation of all species (Fig. 1). Restricted-range species and Chiroptera were effective indicator groups, performing similar to the ideal model (Tukey's test, q value  = 1.95 and 1.89, respectively; p>0.01– Fig. 1 and Table S1).

As expected, some indicator groups performed substantially better than others. In the Cerrado, indicator groups represented *ca.* 78% (±2.4% SD) and 88% (±1.4% SD) of all species. In the Atlantic Forest, indicator groups represented *ca.* 80% (±2.0% SD) and 87% (±4.3% SD) of all species (Fig. 1). The number of sites required for representing all species of each indicator group ranged from eight (for Carnivora) to 50 (for all species), in the Cerrado; and nine (for Carnivora) to 60 (for all species), in the Atlantic Forest.

### Indicator group performance in representing target groups

**Figure 2:**
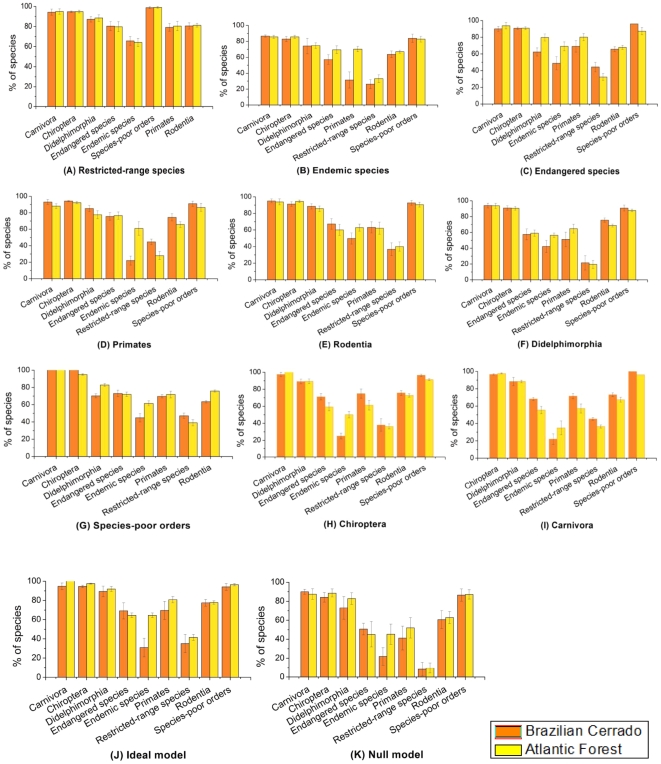
Effectiveness and consistency of each indicator group to represent mammal taxonomic groups in the Cerrado and the Atlantic Forest Biodiversity Hotspots. Graphs indicate how much diversity each indicator group (A–K) captured from each mammal taxonomic group in both Biodiversity Hotspots. Effectiveness and consistency were measured as the percentage of all species included in eight (Cerrado) and nine (Atlantic Forest) sites selected to protected all species of each indicator groups. Bars heights represent means of 20 reserve-selection analyses, error bars represent standard deviations. The ideal model and the null model stand for the result of sites selected based on all species pooled together and random species sets, respectively.

Some indicator groups also performed better than others in representing target species. Again, restricted-range species was the best indicator group being more effective in representing all target species than groups randomly assorted. The performance of restricted-range species, varying from 66% (±4.3% SD) to 99% (±1.0% SD) in the Cerrado, and from 64% (±3.2% SD) to 99% (±1.0% SD) in the Atlantic Forest was statistically equal to the ideal model: 69% ±8.4% SD in the Cerrado, and 65% ±2.3% SD in the Atlantic Forest (q value  = 1.89, p>0.01, Fig. 2). Random species sets captured 8–90% of target species in the Cerrado, and 35–100% in the Atlantic Forest. Contrastingly, selecting sites based on endemic species provided less species representation than selecting sites based on random species sets.

Some indicator groups were also much better represented than others. The performance of indicator groups in representing Carnivora, Chiroptera, Didelphimorphia and species-poor orders ranged from 85% (±3.5% SD) to 100% (±0.0% SD) in the Cerrado, and from 86% (±3.2% SD) to 100% (±0% SD) in the Atlantic Forest (but some groups proved to be inefficient; Fig. 2). Although some groups represented a relatively large percentage of Carnivora, Chiroptera, Didelphimorphia and species-poor orders, they also represented a rather low percentage of restricted-range and endemic species. Despite some indicator groups were more effective in representing restricted-range and endemic species than random sets of species, their performances were relatively low. They represent between 21% (±9.1% SD) and 47% (±3.2% SD) of restricted-range species, and 42% (±6.7% SD) and 50% (±6.6% SD) of endemic species, in the Cerrado; and between 19% (±5.1% SD) and 42%(±3.2% SD) of restricted-range species and 50% (±3.7% SD) and 63% (±4.3% SD) of endemic species in the Atlantic Forest (Fig. 2).

### Consistency of indicator groups

Only restricted-range species and Chiroptera performed consistently well in both Biodiversity Hotspots. On average, sites selected based on the distribution of restricted-range species captured 88% (±1.4% SD) of overall diversity in the Cerrado and 87% (±1.9% SD) in the Atlantic Forest. Sites selected to represent Chiroptera captured 87% (±3.4% SD) of mammal species in the Cerrado and 84% (±0.9% SD) in the Atlantic Forest (Fig.1).

When considering the representation of target groups, only restricted-range species was consistent (Fig.2, Table S1), with average representation between 64% (±4.2% SD) and 99% (±1.0% SD). Note that all other groups were inconsistent in the representation of endemic species.

## Discussion

We show that priority sites selected based on indicator groups can include a large percentage of overall mammal diversity. Further, restricted-range species conveyed effective and consistent representation of mammals in both Biodiversity Hotspots. Some studies have reached similar conclusions [11], [21], [24], whereas others have demonstrated the inefficiency of indicator groups in representing restricted-range species, in particular [21]. Although still controversial (see Lawler et al. [21]) some authors argue that effective surrogacy requires the selection of large tracts of land so that most target species will be represented [30]. Contrastingly, here we showed that good surrogates required the selection of relatively small percentages (1.2–1.9%) of the Biodiversity Hotspots to outperform sites selected based on random species sets and even better than the ideal model.

Despite these relatively optimistic results, we must highlight a critical shortcoming here: first, these results hold for a very small and simple conservation goal of representing species in at least three grid cells. This is worryingly given that it shows that maintenance costs increase significantly with the implementation of protected areas to ensure the maintenance of species populations in the long term. Second, we used range maps as a proxy to species' geographic distribution. It is known that these maps tend to overestimate species' distribution [13] and then increase overall effectiveness of indicator groups whose distribution was based on range maps. One possible solution would be to model the distribution of all species using one or several combined methods for species distribution modeling currently available [31]. However, these models are full of uncertainties, most of which come from the algorithm used to model the species distribution and from the climate model applied to associate species' occurrence to climatic data, which could undermine conservation planning [32]–[36]. Uncertainties are also geographically structure [32], so that some regions of the world are particularly problematic for using such approach. Moreover, as we are not proposing the establishment of protected areas but rather suggesting that the use of some indicator groups are likely to be a shortcut for conservation assessment, using range maps still figure as a possible solution for investigating indicator group effectiveness and consistency, as demonstrated by several other studies [5], [11], [12], [21]–[24].

An outstanding performance of restricted-range species is linked its unique scattered pattern of species' geographic distribution and the number of species composing this group. Restricted-range species, having small and scattered distributions cover a wide range of environmental conditions and spatial heterogeneity, co-occurring with habitat-specialized species, and leading to more complementary sets (i.e. higher beta-diversity) than any other group. This has been hypothesized also by Lawler et al. [11] and Larsen et al. [24], who reached similar conclusions (see also Pinto et al. [22] and Loyola et al. [23]). Alternatively, there might be another explanation for such a high performance of restricted-range species. For obvious reasons, when we evaluated the performance of restricted-range species in representing any other group, we removed these species of that particular group (otherwise we would clearly overestimate the effectiveness of the indicator). However, no group is capable of representing range-restricted species quite well – which implies an advantage to the later. Following this reasoning, every indicator group would need to represent some (if not all) of restricted-range species which are spread through all mammal orders. This means that restricted-range species, being difficult to be captured, might decrease the average representation of each indicator group, i.e. when one uses restricted-range species as an indicator group, be default, one protects one of the hardest groups to represent in the studied Biodiversity Hotspots. This explanation does not rule out the first one, instead, it helps us to further understand why restricted-range species had such a good performance in this study.

Endemic and endangered species are also important targets for continental and global conservation efforts [37]–[39] because they often have small populations and few sites still available for conservation. Differently from previously observed (e.g. Lamoreux et al. [29]), we showed that only restricted-range species have patterns of distribution geographic congruent with all others species. Hence, only this group provides efficiency and consistency in all studied regions; the use of these species is crucial to capture groups of species of great ecological interest, such as endangered species and endemic species. We also showed that endemic species were not good indicator groups. This happens partially because of the distribution patterns of these species in the Cerrado and Atlantic Forest; these local endemic species have clumped spatial distributions in these regions and therefore do not capture the multiple environmental gradients covered by restricted-range species.

Finally, it is worth noting that our analyses evaluated the effectiveness of indicator groups in representing only species richness as our measure of biodiversity, and thus do not incorporate other important aspects such as the persistence of biodiversity, as well as functional and phylogenetic diversity, for example [40], [41]. For now, we can generalize studies with indicator groups only if the group is composed by specie having restricted geographical distribution, which would increase our predictive ability to represent species across different sites. Restricted-range species is the single group which seems to achieve the standards of an effective and consistent surrogate for representing threatened and endemic species in two top-ranked Biodiversity Hotspots. Conservation actions relying on other taxonomic groups are supposed to fail to protect the imperiled fauna of such important and unique regions of the globe.

## Materials and Methods

### Data and scope of study

We superimposed a grid system with cells of 0.5° latitude x 0.5° longitude (*ca.* 52.5 km side at the Equator) to two top-ranked Biodiversity Hotspot, obtaining a network of 678 grid cells for the Brazilian Cerrado and 469 grid cells for the Atlantic Forest. We clipped extent of occurrence maps (available at http://www.iucnredlist.org/technical-documents/spatial-data) for 392 terrestrial mammal species inhabiting these Biodiversity Hotspots and associated them with our grid system.

The Cerrado is the second largest Brazilian domain, extending over an area of 2.036.448 km^2^, 23.92% of Brazilian territory [42]. The Atlantic Forest biome had an original area of 1.233.875 km^2^ of which only 11.4–16% remains [43], with remnants is present mostly in the Brazilian territory, but including also the east parts of Paraguay and the province of Misiones, in Argentina. We chose these Biodiversity Hotspots as our case study for some reasons: (1) they are very different in respect to their inhabiting fauna and flora, geological aspect (including soils and relief), and natural vegetation cover; the Atlantic Forest is mostly composed of forest ecosystems whereas the Cerrado is a vastly tropical savanna-like ecoregion [44], (2) although they figure as Biodiversity Hotspots, they have received little attention respective to the establishment of protected areas in Brazil [45], (3) they are severely threatened by agriculture and cattle ranching expansion [46], [47], and (4) they require urgent conservation actions, figuring as regions that could provide cost-effective actions in a global context [48]–[50].

We divided mammals into nine potentially indicator groups, as follow: the orders Carnivora, Chiroptera, Primates, Rodentia, and Didelphimorphia, species-poor orders [i.e. those with less than 17 species (Cetartiodactyla, Cingulata, Lagomorpha, Perissodactyla, Pilosa)], threatened species, endemic species and restricted-range species (Table 1). Threatened species were those classified as ‘vulnerable’, ‘endangered’ and ‘critically endangered’ according to IUCN (2010). We defined restricted-range species as the 10% of the species with the smallest number of occupied gird cells in each Biodiversity Hotspot. Note that species with relatively small global range sizes might be widely distributed in our study and that species with relatively large global ranges might have locally restricted distributions in the studied Biodiversity Hotspots (see also Lawler & White [11]).

**Table 1:** The number of species and sites required to maximize the representation of all species of each indicator group and all mammal species in the Brazilian Cerrado and the Atlantic Forest Biodiversity Hotspots.

	Number of species		Number of sites required to represent all species	
Indicator groups	Brazilian	Atlantic	Brazilian	Atlantic
	Cerrado	Forest	Cerrado	Forest
Carnivora	21	22	8	9
Chiroptera	109	98	21	15
Didelphimorphia	31	31	14	15
Endangered species	21	30	21	27
Endemic species	17	48	27	34
Primates	30	25	18	20
Restricted-range species	32	36	43	51
Rodentia	94	113	38	48
Species-poor orders	23	23	10	12
All species	308	312	50	60

### Evaluating the performance and consistency of indicator groups

We used two approaches to evaluate the performance of indicator groups in the Cerrado and the Atlantic Forest. Initially, we searched for the smallest set of grid cells needed to represent all species of each indicator group (the so-called ‘minimum set coverage problem’) [51]. We considered a satisfactory solution that in which each species occurred in at least three grid cells. This representation goal (occurrence in at least three grid cells) stands as a proxy for enhancing species persistence when no information about population viability is available or when a high number of species is considered. Among all potential indicator groups, the order Carnivora needed the least number of grid cells to represent their own species (eight grid cells in the Cerrado and nine grid cells in Atlantic Forest) (Table 1).

Later, we searched for the 20 best sets of sites able to maximize the representation of each indicator group within eight and nine grid cells (in the Cerrado and Atlantic Forest, respectively) –a conservation problem known as the ‘maximal representation problem’ [52]. In this case, we found the best spatial solutions for representing the maximum number of species for each group, with the restriction that these solutions could not exceed eight grid cells in the Cerrado and nine grids in the Atlantic Forest. This was necessary so that the effectiveness of the indicators (in terms of the percentage of represented diversity) could be compared without biases related to the number of sites covered by the group (see also Lawler & White [11]).

Both optimization problems were solved using the simulated annealing algorithm [53], [54], designed with this specific purpose and available in the conservation decision support tool MARXAN [53]. Simulated annealing is an approximate optimization algorithm which starts by drawing one subset of grid cells at random. Then it explores multiple solutions to an objective function, making successive random modifications in initial subset. At each step, the new solution is compared with the previous solution, keeping the best one [53], [54].

The average percentage of target-species representation (i.e. all species except the indicator group being tested) across the Biodiversity Hotspots served as our measure of indicator group performance. For comparison, we run 20 solutions with eight and nine cells (for the Cerrado and the Atlantic Forest, respectively) based on a random set of species and evaluated their effectiveness in representing all species as well as the species of each indicator group. We built these sets to compare whether the performance of indicator groups was higher, similar, or lower than that expected by groups of species randomly assorted (see Larsen et al. [24]). We also ran 20 solutions of eight and nine cells based on the information of all species' distribution. We called these sets ‘ideal models’, i.e. those upon which conservation plans would be ideally based on once all species were considered in the analysis. We then compared the average percentage of representation and consistency of each group indicator group in both Biodiversity Hotspots by two-way Analysis of Variance (ANOVA), in which the Biodiversity Hotspot and the indicator groups were factors and group effectiveness in capturing biodiversity was the response variable. We compared the pairwise performance of each indicator group by the Tukey's posthoc test [11]. We defined effective indicator groups those whose average representation of target species exceeded that obtained by random solutions. We also defined consistent indicator groups as those whose performance in both Biodiversity Hotspots was not statistically different.

Finally, we set the level of significance of our analyses at 1%, given that although sets of solutions for each indicator group were unique, there was high overlap of grid cells tagged as important, reducing the independence of the solutions [11]. Reducing the level of significance to a more conservative value has been accepted as a way to reduce the effects of spatial autocorrelation in spatial patterns when particular methods for controlling this phenomenon is not applicable or simple unnecessary (see Diniz-Filho et al. [55], Kubota et al. [56], and Loyola [57]).

## Supporting Information

Table S1 **The effect of the Biodiversity Hotspot and mammal order or family (in the case of the order Passeriformes) on the effectiveness of indicator groups in representing mammal species in the Brazilian Cerrado and the Atlantic Forest.** Results for the Tukey's test indicate pairwise comparisons of indicator group effectiveness for both Biodiversity Hotspots.
(DOC)

## References

[1] Pimm SL, Russell GJ, Gittleman JL, Brooks TM (1995). The future of biodiversity.. Science.

[2] Vitousek PM, Mooney HA, Lubchenco J, Melillo JM (1997). Human domination of Earth's ecosystems.. Science.

[3] Flynn DFB, Gogol-Prokurat M, Nogeire T, Molinari N, Richers BT (2008). Loss of functional diversity under land use intensification across multiple taxa.. Ecol Lett.

[4] Loreau M, Naeem S, Inchausti P, Bengtsson J, Grime JP (2001). Biodiversity and Ecosystem Functioning: Current Knowledge and Future Challenges.. Science.

[5] Rodrigues ASL, Brooks TM (2007). Shortcuts for biodiversity conservation planning: the effectiveness of surrogates.. Annu Rev Ecol Evol S.

[6] Loucks C, Ricketts TH, Naidoo R, Lamoreux J, Hoekstra J (2008). Explaining the global pattern of protected area coverage: relative importance of vertebrate biodiversity, human activities and agricultural suitability.. J Biogeogr.

[7] Margules CR, Pressey RL (2000). Systematic conservation planning.. Nature.

[8] Pressey R, Humphries C, Margules C, Vane-Wright R, Williams P (1993). Beyond opportunism: key principles for systematic reserve selection.. Trends Ecol Evol.

[9] Purvis A, Hector A (2000). Getting the measure of biodiversity.. Nature.

[10] Carbayo F, Marques AC (2011). The costs of describing the entire animal kingdom.. Trends Ecol Evol.

[11] Lawler JJ, White D (2008). Assessing the mechanisms behind successful surrogates for biodiversity in conservation planning.. Anim Conserv.

[12] Grantham HS, Pressey RL, Wells JA, Beattie AJ (2010). Effectiveness of Biodiversity Surrogates for Conservation Panning: Different Measures of Effectiveness Generate a Kaleidoscope of Variation.. PLoS ONE.

[13] Rondinini C, Wilson KA, Boitani L, Grantham H, Possingham HP (2006). Tradeoffs of different types of species occurrence data for use in systematic conservation planning.. Ecol Lett.

[14] Margules CR, Sarkar S (2007). Systematic Conservation Planning..

[15] Andelman SJ, Fagan WF (2000). Umbrellas and flagships: efficient conservation surrogates or expensive mistakes?. P Natl Acad Sci USA.

[16] Mace G, Possingham HP, Leader-Williams N, Macdonald DW, Service K (2007). Prioritizing choices in conservation.. Key topics in conservation biology.

[17] Faith DP, Walker PA (1996a). Environmental diversity: on the best-possible use of surrogate data for assessing the relative biodiversity of sets of areas.. Biodivers Conserv.

[18] Faith DP, Walker PA (1996b). How do indicator groups provide information about the relative biodiversity of different sets of areas? on hotspots, complementarity and pattern-based approaches.. Biodivers Lett.

[19] Sarkar S, Justus J, Fuller T, Kelley C, Garson J (2005). Effectiveness of environmental surrogates for the selection of conservation area networks.. Conserv Biol.

[20] Trakhtenbrot A, Kadmon R (2005). Environmental cluster analysis as a tool for selecting complementary networks of conservation sites.. Ecol Appl.

[21] Lawler JJ, White D, Sifneos JC, Master LL (2003). Rare species and the use of indicator groups for conservation planning.. Conserv Biol.

[22] Pinto MP, Diniz-Filho JAF, Bini LM, Blamires D, Rangel TFLVB (2008). Biodiversity surrogate groups and conservation priority areas: birds of the Brazilian Cerrado.. Divers Distrib.

[23] Loyola RD, Kubota U, Lewinsohn TM (2007). Endemic vertebrates are the most effective surrogates for identifying conservation priorities among Brazilian ecoregions.. Divers Distrib.

[24] Larsen FW, Bladt J, Rahbek C (2009). Indicator taxa revisited: useful for conservation planning?. Divers Distrib.

[25] Bani L, Massimino Bottoni DL, Massa R (2006). A multiscale method for selecting indicator species and priority conservation areas: a case study for broadleaved forests in Lombardy, Italy.. Conserv Biol.

[26] Chiarucci A, D'auria F, Bonini I (2007). Is vascular plant species diversity a predictor of bryophyte species diversity in Mediterranean forest?. Biodivers Conserv.

[27] Schmit JP, Mueller GM, Leacock PR, Mata JL, Wu QF (2005). Assessment of tree species richness as a surrogate for macrofungal species richness.. Biol Conserv.

[28] Lewandowski AS, Noss RF, Parsons DR (2010). The Effectiveness of Surrogate Taxa for the Representation of Biodiversity.. Conserv Biol.

[29] Lamoreux JF, Morrison JC, Ricketts TH, Olson DM, Dinerstein E (2006). Global tests of biodiversity concordance and the importance of endemism.. Nature.

[30] Howard PC, Viskanic P, Davenport TRB, Kigenyi FW, Baltzer M (1998). Complementarity and the use of indicator groups for reserve selection in Uganda.. Nature.

[31] Araújo MB, New M (2007). Ensemble forecasting of species distributions.. Trends Ecol Evol.

[32] Diniz-Filho JAF, Bini LM, Rangel TF, Loyola RD, Hof C (2009). Partitioning and mapping uncertainties in ensembles of forecasts of species turnover under climate change.. Ecography.

[33] Diniz JAF, de Oliveira G, Bini LM, Loyola RD, Nabout JC (2009). Conservation biogeography and climate change in the Brazilian Cerrado.. Nat Conservacao.

[34] Diniz JAF, Nabout JC, Bini LM, Loyola RD, Rangel TF (2010). Ensemble forecasting shifts in climatically suitable areas for *Tropidacris cristata* (Orthoptera : Acridoidea: Romaleidae).. Insect Conserv Diver.

[35] Wilson KA, Westphal MI, Possingham HP, Elith J (2005). Sensitivity of conservation planning to different approaches to using predicted species distribution data.. Biol Conserv.

[36] Loiselle BA, Howell CA, Graham CH, Goerck JM, Brooks T (2003). Avoiding pitfalls of using species distribution models in conservation planning.. Conserv Biol.

[37] Myers N, Mittermeier RA, Mittermeier CG, Fonseca GAB, Kent J (2000). Biodiversity hotspots for conservation priorities.. Nature.

[38] Stattersfield AJ, Crosby MJ, Long AJ, Wege DC (1998). Endemic Bird Areas of the World: Priorities for Biodiversity Conservation..

[39] Loyola RD, Kubota U, Fonseca GAB, Lewinsohn TM (2009). Key Neotropical ecoregions for conservation of terrestrial vertebrates.. Biodivers Conserv.

[40] Devictor V, Mouillot D, Meynard C, Jiguet F, Thuiller W (2010). Spatial mismatch and congruence between taxonomic, phylogenetic and functional diversity: the need for integrative conservation strategies in a changing world.. Ecol Lett.

[41] Carvalho RA, Cianciaruso MV, Trindade-Filho J, Sagnori MD, Loyola RD (2010). Drafting a blueprint for functional and phylogenetic diversity conservation in the Brazilian Cerrado.. Nat Conservacao.

[42] Oliveira PS, Marquis RJ, (Orgs.) (2002). The Cerrados of Brazil: Ecology and Natural History of a Neotropical Savanna..

[43] Ribeiro MC, Metzger JP, Martensen AC, Ponzoni FJ, Hirota MM (2009). The Brazilian Atlantic Forest: How much is left, and how is the remaining forest distributed? Implications for conservation.. Biol Conserv.

[44] Rizzini CT (1997). Tratado de fitogeografia do Brasil..

[45] Rylands AB, Brandon K (2005). Brazilian protected areas.. Conserv Biol.

[46] Diniz-Filho JAF, Bini LM, Pinto MP, Terrilile LC, Oliveira G (2008). Conservation planning: a macroecological approach using the endemic terrestrial vertebrates of the Braziilan Cerrado.. Oryx.

[47] Dobrovolski R, Diniz-Filho JAF, Loyola RD, De Marco P (2011). Agricultural expansion and the fate of global conservation priorities.. Biodivers Conserv.

[48] Jenkins CN, Alves MAS, Pimm SL (2010). Avian conservation priorities in a top-ranked biodiversity hotspot.. Biol Conserv.

[49] Becker CG, Loyola RD, Haddad CFB, Zamudio KR (2010). Integrating species life-history traits and patterns of deforestation in amphibian conservation planning.. Divers Distrib.

[50] Loyola RD, Oliveira-Santos LGR, Almeida-Neto M, Nogueira DM, Kubota U (2009). Integrating Economic Costs and Biological Traits into Global Conservation Priorities for Carnivores.. PLoS ONE.

[51] Underhill LG (1994). Optimal and suboptimal reserve selection algorithms.. Biol Conserv.

[52] Church RL, Stoms DM, Davis FW (1996). Reserve selection as a maximal covering location problem.. Biol Conserv.

[53] Possingham HP, Ball I, Andelman SJ, Ferson S, Burgman M (2000). Mathematical methods for identifying representative reserve networks.. Quantitative methods for conservation biology.

[54] Kirkpatrick S, Gelatt CD, Vecchi MP (1983). Optimization by simulated annealing.. Science.

[55] Diniz-Filho JAF, Bini LM, Hawkins BA (2003). Spatial autocorrelation and red herrings in geographical ecology.. Global Ecol Biogeogr.

[56] Kubota U, Loyola RD, Almeida AM, Carvalho DE, Lewinsohn TM (2007). Body size and host range co-determinate the altitudinal distribution of Neotropical tephritid flies.. Global Ecol Biogeogr.

[57] Loyola RD (2009). Broad-scale hypotheses do not account for species richness patterns of Central American mayflies.. The Open Ecology Journal.

