# Supplementary material for: Performance and Consistency of Indicator Groups in Two Biodiversity Hotspots
Source: PLoS One. 2011 May 26;6(5):e19746. doi: 10.1371/journal.pone.0019746 (PMC3102650; doi:10.1371/journal.pone.0019746)
Supplement: Table S1 — The effect of the Biodiversity Hotspot and mammal order or family (in the case of the order Passeriformes) on the effectiveness of indicator groups in representing mammal species in the Brazilian Cerrado and the Atlantic Forest. Results for the Tukey's test indicate pairwise comparisons of indicator group effectiveness for both Biodiversity Hotspots. (DOC) [file pone.0019746.s001.doc]

Table S1. The effect of the Biodiversity Hotspot and mammal order or family (in the case of the order Passeriformes) on the effectiveness of indicator groups in representing mammal species in the Brazilian Cerrado and the Atlantic Forest. Results for the Tukey’s test indicate pairwise comparisons of indicator group effectiveness for both Biodiversity Hotspots.

|  | ANOVA | | | |  | Tukey’s test | | |
| --- | --- | --- | --- | --- | --- | --- | --- | --- |
| Source of variation | Degrees of freedom | Mean Square | F value | P value |  | Mean difference | Q value | P value |
| Biodiversity Hotspot | 1 | 47.78 | 7.08 | 0.008 |  | ― | ― | ― |
| Indicator group | 10 | 8366.76 | 124.05 | 0.001 |  | ― | ― | ― |
| Interaction | 10 | 2047.29 | 30.35 | 0.001 |  | ― | ― | ― |
| Model | 21 | 10461.84 | 73.86 | 0.001 |  | ― | ― | ― |
| Error | 418 | 2819.25 | ― | ― |  | ― | ― | ― |
| Chiroptera * Carnivora | ― | ― | ― | ― |  | 2.075 | 5.053 | 0.017 |
| Didelphimorphia * Carnivora | ― | ― | ― | ― |  | -2.175 | 5.297 | 0.009 |
| Didelphimorphia * Chiroptera | ― | ― | ― | ― |  | -4.25 | 10.351 | 0.001 |
| Speeces-poor orders * Carnivora | ― | ― | ― | ― |  | -1.925 | 4.688 | 0.039 |
| Species-poor orders * Chiroptera | ― | ― | ― | ― |  | -4 | 9.741 | 0.001 |
| Species-poor orders * Didelphimorphia | ― | ― | ― | ― |  | 0.25 | 0.609 | 1 |
| Primates * Carnivora | ― | ― | ― | ― |  | -0.725 | 1.765 | 0.976 |
| Primates * Chiroptera | ― | ― | ― | ― |  | -2.8 | 6.818 | 0.001 |
| Primates * Didelphimorphia | ― | ― | ― | ― |  | 1.45 | 3.531 | 0.309 |
| Primates * Species-poor orders | ― | ― | ― | ― |  | 1.2 | 2.922 | 0.602 |
| Rodentia * Carnivora | ― | ― | ― | ― |  | 0.375 | 0.913 | 0.999 |
| Rodentia * Chiroptera | ― | ― | ― | ― |  | -1.7 | 4.141 | 0.118 |
| Rodentia * Didelphimorphia | ― | ― | ― | ― |  | 2.55 | 6.211 | 0.001 |
| Rodentia * Species-poor orders | ― | ― | ― | ― |  | 2.3 | 5.601 | 0.004 |
| Rodentia * Primates | ― | ― | ― | ― |  | 1.1 | 2.679 | 0.721 |
| Endangered species * Carnivora | ― | ― | ― | ― |  | -0.9 | 2.192 | 0.902 |
| Endangered species * Chiroptera | ― | ― | ― | ― |  | -2.975 | 7.245 | 0.001 |
| Endangered species * Didelphimorphia | ― | ― | ― | ― |  | 1.275 | 3.105 | 0.509 |
| Endangered species * Species-poor orders | ― | ― | ― | ― |  | 1.025 | 2.496 | 0.799 |
| Endangered species * Primates | ― | ― | ― | ― |  | -0.175 | 0.426 | 1 |
| Endangered species * Rodentia | ― | ― | ― | ― |  | -1.275 | 3.105 | 0.509 |
| Restricted-range species * Carnivora | ― | ― | ― | ― |  | 3.65 | 8.889 | 0.001 |
| Restricted-range species * Chiroptera | ― | ― | ― | ― |  | 1.575 | 3.836 | 0.198 |
| Restricted-range species * Didelphimorphia | ― | ― | ― | ― |  | 5.825 | 14.186 | 0.001 |
| Restricted-range species * Species-poor orders | ― | ― | ― | ― |  | 5.575 | 13.577 | 0.001 |
| Restricted-range species * Primates | ― | ― | ― | ― |  | 4.37 | 10.654 | 0.001 |
| Restricted-range species * Rodentia | ― | ― | ― | ― |  | 3.275 | 7.976 | 0.001 |
| Restricted-range species * Endangered species | ― | ― | ― | ― |  | 4.55 | 11.081 | 0.001 |
| Endemic species * Carnivora | ― | ― | ― | ― |  | -10.225 | 24.901 | 0 |
| Endemic species * Chiroptera | ― | ― | ― | ― |  | -12.3 | 29.954 | 0.001 |
| Endemic species * Didelphimorphia | ― | ― | ― | ― |  | -8.05 | 19.604 | 0.001 |
| Endemic species* Species-poor orders | ― | ― | ― | ― |  | -8.3 | 20.213 | 0 |
| Endemic species * Primates | ― | ― | ― | ― |  | -9.5 | 23.135 | 0 |
| Endemic species * Rodentia | ― | ― | ― | ― |  | -10.6 | 25.814 | 0.001 |
| Endemic species * Endangered species | ― | ― | ― | ― |  | -9.325 | 22.709 | 0 |
| Endemic species * Restricted-range species | ― | ― | ― | ― |  | -13.875 | 33.789 | 0 |
| All species * Carnivora | ― | ― | ― | ― |  | 2.875 | 7.001 | 0.001 |
| All species * Chiroptera | ― | ― | ― | ― |  | 0.8 | 1.948 | 0.953 |
| All species * Didelphimorphia | ― | ― | ― | ― |  | 5.05 | 12.298 | 0.001 |
| All species * Species-poor orders | ― | ― | ― | ― |  | 4.8 | 11.689 | 0.001 |
| All species * Primates | ― | ― | ― | ― |  | 3.6 | 8.767 | 0.001 |
| All species * Rodentia | ― | ― | ― | ― |  | 2.5 | 6.088 | 0.001 |
| All species * Endangered species | ― | ― | ― | ― |  | 3.775 | 9.193 | 0.001 |
| All species * Restricted´range species. Restrita | ― | ― | ― | ― |  | -0.775 | 1.887 | 0.962 |
| All species * Endemic species | ― | ― | ― | ― |  | 13.1 | 31.902 | 0.001 |
| Random * Carnivora | ― | ― | ― | ― |  | -9.7 | 23.622 | 0 |
| Random * Chiroptera | ― | ― | ― | ― |  | -11.775 | 28.676 | 0.001 |
| Random * Didelphimorphia | ― | ― | ― | ― |  | -7.525 | 18.326 | 0.001 |
| Random * Speeces-poor orders | ― | ― | ― | ― |  | -7.775 | 18.934 | 0.001 |
| Random * Primates | ― | ― | ― | ― |  | -8.975 | 21.857 | 0 |
| Random * Rodentia | ― | ― | ― | ― |  | -10.075 | 24.535 | 0 |
| Random * Endangered species | ― | ― | ― | ― |  | -8.8 | 21.431 | 0 |
| Random * Restricted-range species | ― | ― | ― | ― |  | -13.35 | 32.511 | 0.001 |
| Random * Endemic species | ― | ― | ― | ― |  | 0.525 | 1.278 | 0.998 |
| Random * All species | ― | ― | ― | ― |  | -12.575 | 30.624 | 0.001 |
